# The role of Toll-like receptor 9 in a murine model of *Cryptococcus gattii* infection

**DOI:** 10.1038/s41598-021-80959-5

**Published:** 2021-01-14

**Authors:** Elias Barbosa da Silva-Junior, Luan Firmino-Cruz, Joyce Cristina Guimarães-de-Oliveira, Juliana Valente Rodrigues De-Medeiros, Danielle de Oliveira Nascimento, Matheus Freire-de-Lima, Lycia de Brito-Gitirana, Alexandre Morrot, Jose Osvaldo Previato, Lucia Mendonça-Previato, Debora Decote-Ricardo, Herbert Leonel de Matos Guedes, Celio Geraldo Freire-de-Lima

**Affiliations:** 1 Instituto de Biofísica Carlos Chagas Filho, Universidade Federal do Rio de Janeiro, Rio de Janeiro, 21941-900 Brazil; 2 Instituto Oswaldo Cruz, FIOCRUZ, Rio de Janeiro, 21045-900 Brazil; 3 Instituto de Veterinária, Universidade Federal Rural do Rio de Janeiro, Seropédica, 23890-000 Brazil; 4 Instituto de Ciências Biomédicas, Universidade Federal do Rio de Janeiro, Rio de Janeiro, 21941-900 Brazil; 5 Faculdade de Medicina, Universidade Federal do Rio de Janeiro, Rio de Janeiro, 21941-900 Brazil

**Keywords:** Microbiology, Fungi, Fungal pathogenesis

## Abstract

Toll-like receptor 9 (TLR9) is crucial to the host immune response against fungi, such as *Candida albicans*, *Aspergillus fumigatus* and *Cryptococcus neoformans*, but its importance in *Cryptococcus gattii* infection is unknown. Our study aimed to understand the role of TLR9 during the course of experimental *C. gattii* infection in vivo, considering that the cryptococcal DNA interaction with the receptor could contribute to host immunity even in an extremely susceptible model. We inoculated C57BL/6 (WT) and TLR9 knock-out (TLR9^−/−^) mice intratracheally with 10^4^ *C. gattii* yeast cells. TLR9^−/−^ mice had a higher mortality rate compared to WT mice and more yeast cells that had abnormal size, known as titan cells, in the lungs. TLR9^−/−^ mice also had a greater number of CFUs in the spleen and brain than WT mice, in addition to having lower levels of IFN-γ and IL-17 in the lung. With these markers of aggressive cryptococcosis, we can state that TLR9^−/−^ mice are more susceptible to *C. gattii*, probably due to a mechanism associated with the decrease of a Th1 and Th17-type immune response that promotes the formation of titan cells in the lungs. Therefore, our results indicate the participation of TLR9 in murine resistance to *C. gattii* infection.

## Introduction

Cryptococcosis is a worldwide distributed invasive mycosis caused by the pathogenic fungi *Cryptococcus* spp. Of the 37 characterized species, only *C. neoformans* and *C. gattii* are considered the etiological agents of cryptococcosis. *Cryptococcus* spp. are the only encapsulated fungi capable of causing disease in humans^1–3^. The polysaccharide capsule that covers these yeasts represents one of their most important virulence factors and has high immunomodulatory potential^3–6^. This capsule consists mainly of glucuronoxylomannan (GXM) and galactoxylomannan (GalXM), which can be released at all times into host tissues^3,5–7^. Although the polysaccharide capsule may be the principal virulence factor for host susceptibility to both *C. neoformans* and *C. gattii*, other virulence factors are crucial for the establishment of infection, such as the formation of titan cells, melanin production, growth at 37 °C, phospholipase synthesis, ureases and vesicle release^6,8–15^.

In recent decades, cryptococcosis has been of global importance after a North American outbreak, as well as its increasing incidence in countries from Africa, Europe and South America. Moreover, as the number of elderly, immunocompromised and individuals with biologic immune suppression due to organ transplants grows so too does the proportion of the population susceptible to cryptococcal infection^3,16–20^. The presence of *C. gattii* in the environment is well documented and described^18,19,21,22^. Cryptococcosis has reported cases on all continents, although the majority of cases are reported in USA and Canada^18^. The pathogenicity in humans and animals is known in countries from Oceania, Europe, the Americas and some regions of Asia^17,18,21^. However, the incidence zone of cryptococcosis is expanding around the world^21,22^.

The disease can occur after inhalation of infectious forms of the fungus, which can be basidiospores or desiccated yeast cells, carried through the air that become deposited in the pulmonary alveolus. This is considered a primary pulmonary infection, which may lead to a disseminated infection^17^. The initial immune response occurs through the recognition of *C. gattii* by alveolar macrophages, followed by the activation and recruitment of inflammatory cells through cytokines and chemokines. However, the fungus has several escape mechanisms and can make successive budding divisions, culminating in phagosomes replete with cryptococcal cells, in addition to large amounts of GXM and GalXM^3,5,21^. Recently, some studies have shown that, in advanced stages of infection, *C. neoformans *is able to abnormally enlarge its own cells, which are known as titan cells^23–26^. Abnormal cell size is described as a vital mechanism of protection against phagocytosis and is associated with pathways that result in a thick polysaccharide capsule, a thickened cell wall and melanin production^19,20^. Although cryptococcosis is a respiratory disease, causing cryptococcal pneumonia, fungal tropism for the central nervous system (CNS) can occur^3,14^. The spread of *Cryptococcus* to the CNS leads to the development of cryptococcal meningitis, described as the principal cause of mortality during infections by *C. gattii*^3,21^.

The components of the innate immune system act as first responders for the detection and clearance of infectious agents. Classically, innate immune receptors recognize pathogen-associated molecular patterns (PAMPs). These receptors can be divided into three major groups: NOD-like receptors (NLRs), RIG-like receptors (RLRs) and Toll-like receptors (TLRs). TLRs are a family of signaling receptors on membranes^27^. They are widely studied and play essential roles in innate immunity, especially in tissue inflammation and in the recognition of microorganisms, such as viruses, bacteria and fungi. Many studies have shown the importance of TLR9, a receptor of the TLR family, in fungal infections, mainly in models of infection by *Aspergillus fumigatus*^28^, *Candida albicans*^29^ and *Cryptococcus neoformans*^30,31^. TLR9 is found in endosomal vesicles and capable of recognizing fractions of non-methylated DNA, which are common in viruses, prokaryotes and some protozoa, such as those of the genus *Leishmania*^32,33^. TLR9 is located in endoplasmic reticulum and is translocated to the lysosome and Golgi complex after direct interaction with CpG motifs present in single-stranded DNA (ssDNA). The stimulation triggers intracellular signaling dependent on recruitment and interaction with MyD88, leading to the activation of macrophages, dendritic cells (DCs) and B cells, and the production of cytokines, chemokines, and immunoglobulins^34^. Subsequently, cytokines produced by DCs, such as IL-12 or IL-6, induce the differentiation of naive T cells into Th1 and Th17^35^, respectively. Studies using murine models have demonstrated that the control of spread and infection by *Cryptococcus* requires a cellular and molecular adaptive response, involving the Th1 and Th17-type immune response^36–39^. TLR9 signaling is crucial for the generation of adaptive immune protection against *C. neoformans* infection^38,40^. *C. neoformans* DNA is capable of activating TLR9 in C57BL/6 mice during experimental infection. Bone marrow dendritic cells (BM-DCs) produced high amounts of IL-12p40 and expressed more CD40 on their surface when co-cultured with *C. neoformans* lysate, and the response was abolished by treatment with DNase. The same phenomenon could be observed when the cells were activated with TLR9 ligands^41^. However, studies regarding the adaptive response against *C. gattii* are scarce, especially involving the protective Th1 and Th17-type immune response and TLR9.

The objective of the current study was to investigate the role of TLR9 in a murine model of experimental *C. gattii* infection. Our findings reveal that TLR9 is important for the survival of C57BL/6 (WT) mice infected with 10^4^ yeast cells of *C. gattii*. TLR9-deficient (TLR9^−/−^) mice have a higher fungal burden in the organs and a higher frequency of mortality compared to infected WT animals. In addition, TLR9^−/−^ mice produce fewer protective cytokines, such as IL-17 and IFN-γ. Thus, our results provide a better understanding of the immunomodulatory role of TLR9 in experimental infection caused by *C. gattii.*

## Results

### TLR9^−/−^ mice are more susceptible to ***C. gattii*** infection than WT mice

**Figure 1:**
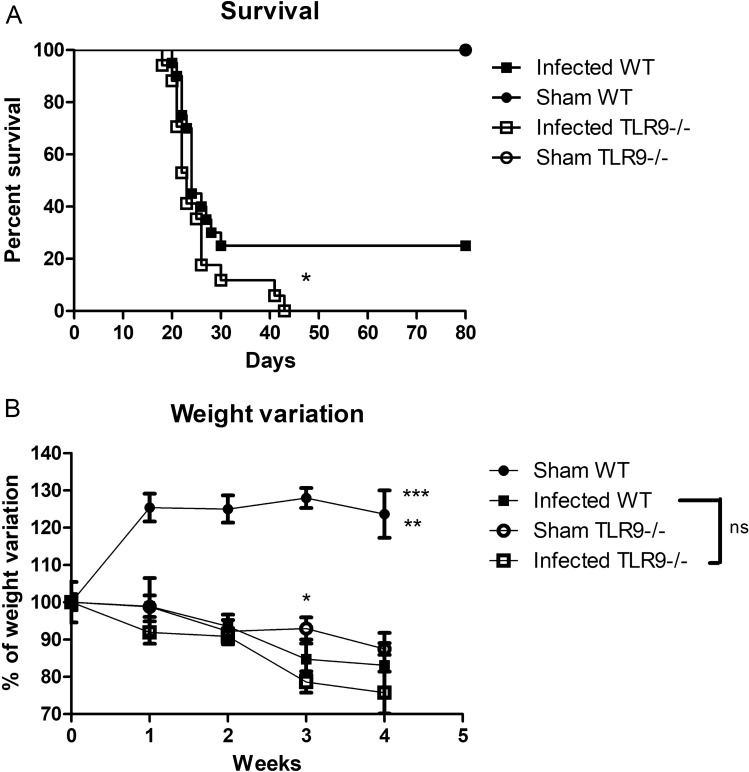
Analysis of susceptibility of TLR9^−/−^ and WT mice to *C. gattii* infection. (**A**) Survival curves of infected C57BL/6 WT (n = 20) and TLR9^−/−^ (n = 17) mice. Mice were infected with 10^4^ *C. gattii* R265 belonging to serotype IIa. Sham WT (n = 6) and TLR9^−/−^ (n = 6) mice given with vehicle (PBS) only were used as controls. Log-rank test (Mantel-Cox test): *P ≤ 0.05. (**B**) The percentage change in weight of infected WT (n = 11) and TLR9^−/−^ (n = 10) mice, as well as the Sham groups (Sham WT and Sham TLR9^−/−^) (n = 5 per group), was determined through weekly mass measurements over 28 days (0, 7, 14, 21 and 28 days after inoculation). Black squares represent infected WT mice, black balls represent uninfected WT mice, white squares represent infected TLR9^−/−^ mice, and white balls represent uninfected TLR9^−/−^ animals. One-Way ANOVA (**P ≤ 0.01) and Two-Way ANOVA with Bonferroni posttest (*P ≤ 0.05; ***P ≤ 0.001); ns = not statistically significant. Graphs show the union of the results from 4 similar and independent experiments.

In order to evaluate the susceptibility of C57BL/6 WT and C57BL/6 TLR9^−/−^ mice to *C. gattii* infection, we challenged the animals intratracheally with 10^4^ yeast cells of *C. gattii* (R265), as previously established in our group^42^. Survival and weight variations of infected mice and vehicle (sham) controls (which received only PBS), were evaluated over 4 weeks. As R265 is a hypervirulent cryptococcal strain, we inoculated with this low dose to ensure that the mice survived for at least 2 weeks. Comparing the survival curves of infected animals with the respective Sham group, we observed that both TLR9^−/−^ and WT animals were susceptible to *C. gattii*. However, most WT animals were able to survive for more than 80 days, while there were no live TLR9^−/−^ animals after 43 days post infection (Fig. 1A). We observed from the weight variation curve of the infected TLR9^−/−^ group that there was a progressive decrease, even the same pattern was observed in the infected WT group, with no difference between the groups (Fig. 1B). Curiously, the weight variation curve of Sham TLR9^−/−^ group also indicated weight loss over the weeks (Fig. 1B).

### TLR9^−/−^ mice have higher fungal load in the brain and spleen at 21 days post infection

**Figure 2:**
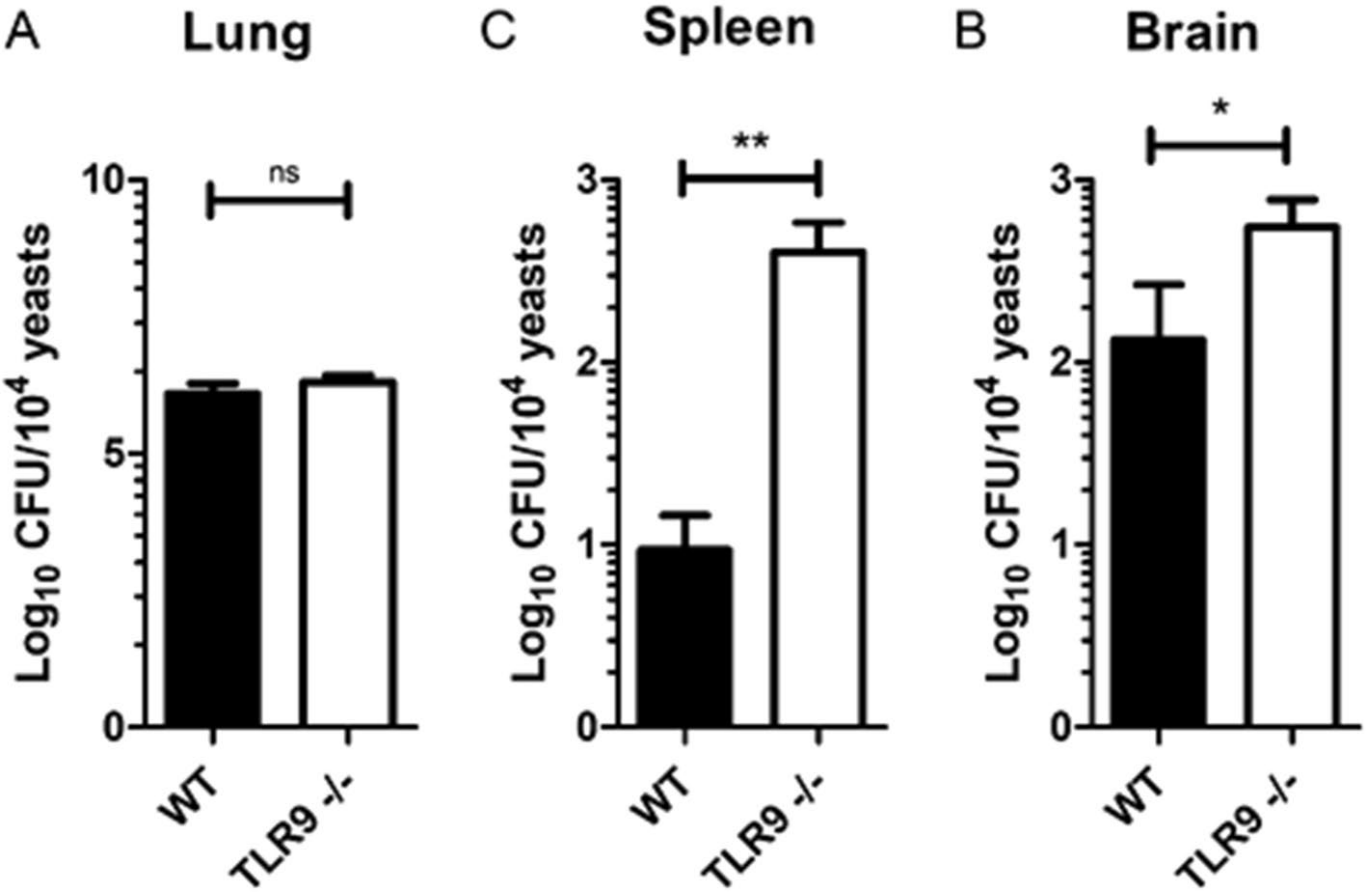
Comparison of CFU counts in different target organs of *C. gattii* infection in WT and TLR9^−/−^ mice. Analysis of fungal load after 21 days of *C. gattii* infection through maceration of tissues taken from WT (n = 6) and TLR9^−/−^ mice (n = 7). (**A**–**C**) The CFUs in the lungs, brain and spleen, respectively. Black bars represent infected C57BL/6 WT mice and white bars represent infected C57BL/6 TLR9^−/−^ mice. Organ homogenates were obtained in 5 mL PBS, the lungs and brain homogenates were then diluted ×10,000 and ×100, respectively, and 50 µL was spread on Petridishes containing Sabouraud's agar. Colonies were counted 72 h after incubation at 37 °C. *CFU* colony forming units. The graphs show a representative result of six similar and independent experiments. Student T-test and Mann–Whitney U test: *P ≤ 0.05; **P ≤ 0.01; ns = not statistically significant.

*Cryptococcus gattii* mainly affects the lungs and CNS, and meningoencephalitis is the most severe and advanced form of cryptococcosis^3,14^. In order to determine the fungal burden and spread of yeast to other tissues, we counted the colony forming units (CFU) of infected WT and TLR9^−/−^ mice at 21 days post-infection. Interestingly, there were no differences in the CFU in the lungs of both infected groups (Fig. 2A). This can be explained by the high lung tissue impairment in the advanced stage of the disease. However, the CFU was higher in the brain (Fig. 2B) and spleen (Fig. 2C) of infected TLR9-deficient mice compared to the infected WT group. The severe spleen and brain impairment of TLR9^−/−^ mice throughout the infection compared to WT mice indicates a greater susceptibility of TLR9-deficient mice to *C. gattii*.

### Infected TLR9^−/−^ mice present higher impairment and yeast dispersion in the lungs compared to WT mice

**Figure 3:**
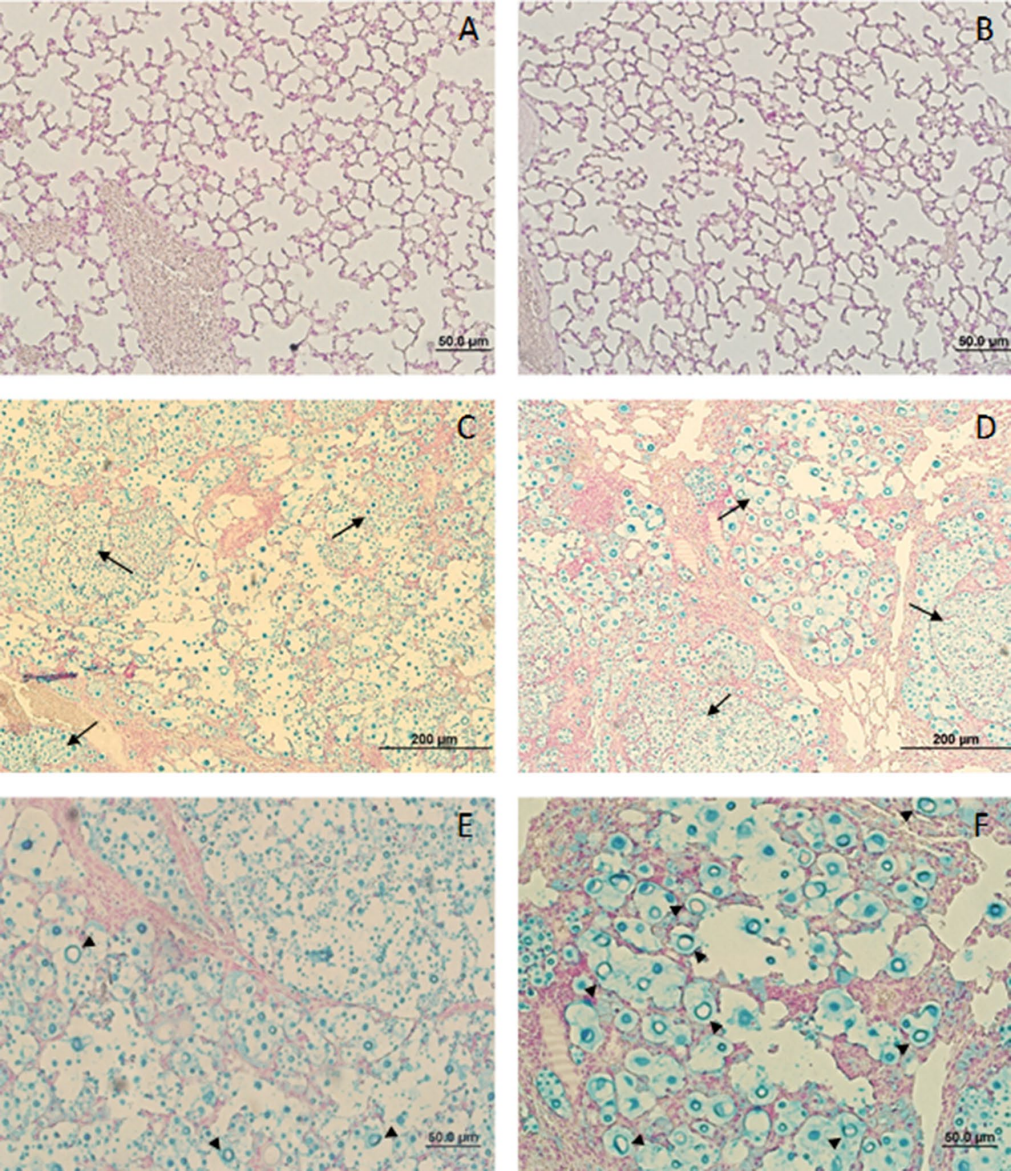
Histological sections of lungs of *C. gattii*-infected WT and TLR9^−/−^ animals. Micrographs of histological sections of lungs obtained from WT and TLR9^−/−^ animals infected with *C. gattii* after 21 days (**C**–**F**) or given sterile PBS (Sham) (**A**, **B**). In the left column, the histological sections of the lungs of WT mice (**A**, **C**, **E**) (n = 3). In the right column, histological sections of the TLR9^−/−^ mice (**B**, **D**, **F**) (n = 4). Magnification, ×40 (**A**–**D**); Magnification ×100 (**E**, **F**). The arrows show normal *C. gattii* yeasts and the arrowheads show *C. gattii* titan cells in the lungs of both WT and TLR9^−/−^ mice. The histological sections were stained with hematoxylin, eosin and Alcian Blue and photographed under a light microscope.

In order to analyze the dispersion and tissue injury caused by *C. gattii* in both WT and TLR9^−/−^ mice after 21 days of infection, histological sections were made and stained with hematoxylin, eosin and Alcian blue. Sham WT lung (Fig. 3A) and Sham TLR9^−/−^ lung (Fig. 3B) histological sections represent the control groups that received PBS intratracheally. *C. gattii* yeast cells were found in the lungs of both infected TLR9^−/−^ and WT groups, as well as a thickening of the alveolar wall, alveolar collapse and increased connective tissue (Fig. 3C–F). However, the large dispersion of yeast, and thickened and collapsed alveolar walls were more frequently observed in the *C. gattii*-infected TLR9-deficient mice compared to WT (Fig. 3C,D). The black arrows indicate *C. gattii* yeast cells located in the lung compartments. In addition, we observed a large number of abnormally sized yeast cells (indicated by black arrowheads), recently described and referred to as *titan cells* in the literature (Fig. 3E,F)^11,23–26,43^. These cells can be produced in response to the host pulmonary environment and play an important role in the local modulation of the host immune response against *C. gattii*.

### Titan cells are more frequent in the lungs of infected TLR9^−/−^ mice

**Figure 4:**
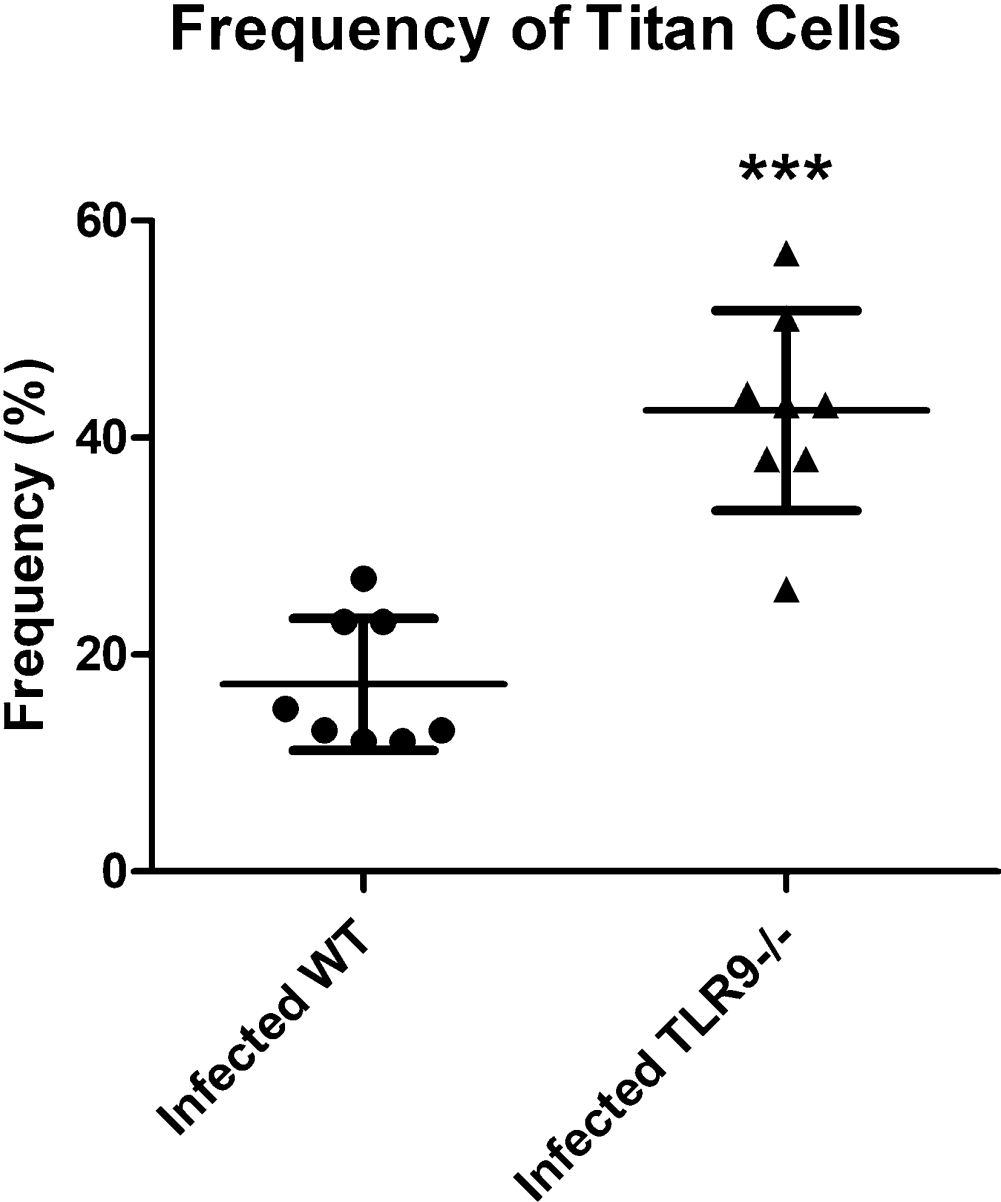
Percentage of titan cells in the lung of *C. gattii*-infected mice. Titan cell (> 10 µm cell size) counts per 100 yeast cells observed in defined quadrants in the histological sections of the lungs of WT (n = 3) and TLR9^−/−^ (n = 4) mice after 21 days of *C. gattii* infection. Each lung (right and left) was divided into two quadrants (4 quadrants for each animal). Titan cells were counted in each quadrant and each result was considered as an absolute value for the respective quadrant. The graphs show a representative result. Student T-test: ***P ≤ 0.0001.

Due to the bigger cell body, titan cells may be able to resist being phagocytosed, leading to host susceptibility to fungal infection^36–38^. We next quantified the number of titan cells (large cell size > 10 µm)^43,44^ in one hundred yeast cells in four quadrants of each lung sample of both the TLR9^−/−^ and WT groups. We counted the cells in histological sections of the lungs stained with hematoxylin, eosin and Alcian Blue using light microscopy. Our results showed that the number of titan cells was higher in the lungs of infected TLR9-deficient mice compared to infected WT mice (Fig. 4). This may be the way that *C. gattii* establishes infection in the host. Studies indicate that the presence of titan cells in tissues infected with *C. neoformans* are associated with higher tissue impairment^23,45^.

Some studies have shown that *C. neoformans* differentiates into cells with altered size and morphology, including the large titan cells, in vitro^26,45^ and in vivo^46^. Regarding *C. gattii* specifically, there is a report demonstrating its differentiation into titan cells in an in vitro studies^26^. The results of the present work are the first to identify the formation of titan cells in an in vivo* C. gattii* experimental infection.

### Infected TLR9^−/−^ mice have a lower production of IFN-γ and IL-17 in comparison to WT mice

**Figure 5:**
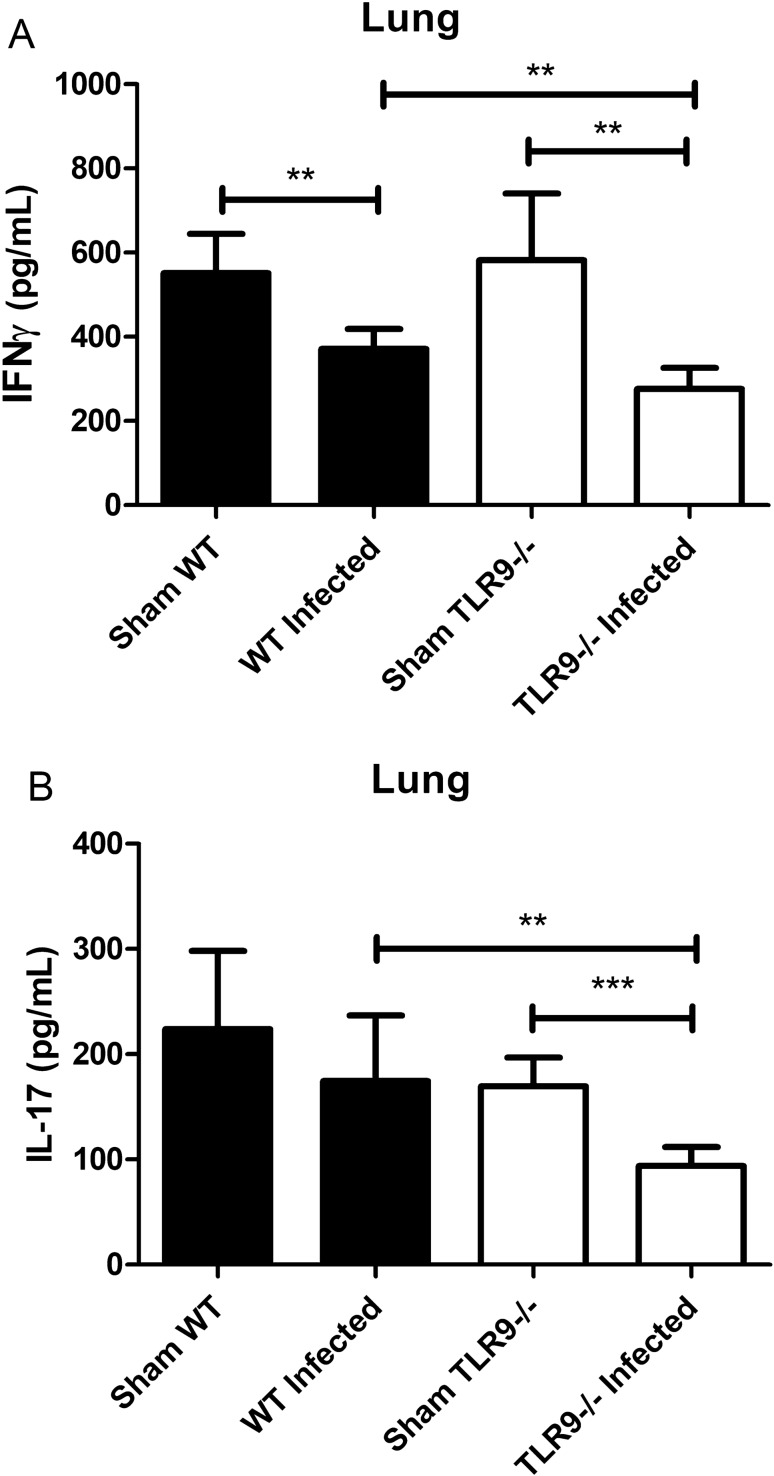
Levels of IFN-γ and IL-17 in total lung homogenates of *C. gattii*-infected mice. Measurement of IFN-γ and IL-17 levels by ELISA of total lung homogenates of WT and TLR9^−/−^ mice, infected with *C. gattii* or given PBS, after 21 days. (**A**) Dosage of IFN-γ and (**B**) IL-17. The graphs show a representative result of two similar and independent experiments. Sham WT (n = 4), Sham TLR9^−/−^ (n = 4), infected WT (n = 6), infected TLR9^−/−^ (n = 7). **P < 0.005; ***P < 0.001; ns = not statistically significant.

TLR9 activates the MyD88 adapter protein and may induce cytokine release and the formation of a Th1 profile. Pro-inflammatory Th1 cytokines are important for the control of microorganisms and, therefore, IFN-γ was the focus of our analysis regarding the effectiveness of combating *C. gattii* infection. IFN-γ production in the lungs decreased in both groups of *C. gattii*-infected mice after 21 days (WT and TLR9^−/−^) compared to their respective control groups (Sham WT and Sham TLR9^−/−^) (Fig. 5A). This result indicates the ability of the pathogen to modulate the host immune response, preventing the establishment of a protective Th1 response during the course of infection. However, it is important to note that TLR9-deficient mice produced even less IFN-γ when compared to infected WT mice, suggesting that TLR9 plays an important role in controlling *C. gattii* infection. The cytokines produced by Th1 are essential for immunity and the cytokines associated with the Th2 profile do not provide protection. It is well described in the literature that the increase in IFN-γ production is related to protection against cryptococcosis, inducing an increase in phagocytic activity and fungicidal activity of phagocytes, favoring the host's response to infection control^47,48^. In an experimental model using an IFN-γ-producing *C. neoformans* strain, the authors observed reduced pulmonary fungal burden, classical macrophage activation, Th1 pulmonary response and IL-17 production^49^.

Several studies have described the importance of the Th17 response in the control of fungal infections, especially in opportunistic fungal models such as *C. neoformans*, *C. albicans* and *A. fumigatus*^35,37,50,51^. IL-17A is important for total lung leukocyte recruitment and accumulation 14 days after infection by *C. neoformans*, as well as the reduction of inflammation and increase in fungal load^50^. Therefore, we analyzed the levels of IL-17, an important Th17 cytokine, 21 days after *C. gattii* infection. Figure 5B shows that IL-17 levels at the primary site of infection were lower in the infected TLR9^−/−^ group compared to the infected WT group. This data may indicate a deficiency of TLR9^−/−^ mice in controlling pathogenesis by a Th17 response during *C. gattii* infection. The difficulty in fighting the microorganism in the lungs could be the way by which the fungus reaches the blood and subsequent rapid spread to the peripheral organs and CNS.

## Discussion

In this work, we evaluated the importance of the receptor TLR9 in murine experimental *C. gattii* infection. A few studies have previously reported that TLR9 activation plays a crucial role in opportunistic mycoses through models of infection with *C. albicans* and *C. neoformans*^29–31^. The results of the present study demonstrate that C57BL/6 mice naturally have difficulty in controlling cryptococcosis (Fig. 1A). Likewise, in a paper published in 2009, Cheng and colleagues observed that around 30% of animals infected with an extremely virulent strain (isolated during an outbreak in North America) remained alive for almost 60 days^42^. However, our study also showed that infected TLR9-deficient mice, which have the C57BL/6 background, almost always died at least a day earlier compared to infected C57BL/6 WT mice. Although TLR9^−/−^ mice have a very similar survival curve to WT mice throughout infection, around 30% of the WT mice remained alive up to 60 days, while all the TLR9^−/−^ mice died by day 43. This result is an indication that TLR9^−/−^ mice are more susceptible to *C. gattii* experimental infection than WT mice.

Figure 1A shows a higher frequency of mortality and a steeper curve for the TLR9^−/−^ group infected with *C. gattii* compared to those of the infected WT group. Susceptibility of TLR9^−/−^ mice to *C. gattii* infection was confirmed in Fig. 2, where the number of CFUs in the tissues typically affected by this pathogen, such as the brain (Fig. 2B) and the spleen (Fig. 2C), reveals that the TLR9-deficient mice experienced more difficulty in controlling the infection. This same phenomenon was observed in experimental models of *C. neoformans* infection in animals that were also deficient in TLR9^31^. However, it is interesting that there are no significant differences in the fungal loads in the lungs, the primary site of infection, after 21 days, between the infected mouse groups, which would have been expected due to the greater susceptibility of the TLR9^−/−^ mice. However, by 21 days of infection, lung damage due to the virulent R265 strain may have been too severe in both mouse strains to permit further discrimination in fungal burden. It is possible that in early stages of the infection there is a difference in the fungal load compared between the infected groups, making it necessary to carry out experiments at other time-points to understand the dynamics of the infection. However, in a model of *C. neoformans* infection in C57BL/6 mice, the difference between experimental times below 21 days of infection was not significant^31^.

The presence of titan cells in our experimental model of cryptococcosis indicates a high susceptibility of C57BL/6 mice to *C. gattii* infection. As recently described, titan cells are linked to the protein kinase A (PKA) pathway in response to the host pulmonary environment^11,52,53^. The PKA pathway is important to other virulence factors, such as the polysaccharide capsule and the dark pigment melanin^13,53^. However, TLR9-deficient mice showed even more tissue impairment and a larger number of titan cells in the lung compared to WT animals (Fig. 4). In the literature, titan cells are described as yeasts that have assumed an abnormal size and that have a strong immunomodulatory role^11,43^. Thus, it is believed that they are cells present in tissues where the damage caused by cryptococcal infection is extreme^46^. The large size may protect the yeast from phagocytosis by alveolar macrophages in the lung, as proposed for *C. neoformans*^25^. Generally, titan cells are > 10 µm in diameter in vitro and in vivo^44^. Phagocytosis by alveolar macrophage is the most critical early response to *Cryptococcus* spp. in the lungs, and Okagaki et al. suggest that the titan cells are vital to the early pulmonary infection caused by *C. neoformans*^24^. The production of *C. neoformans* titan cells was first observed in the lungs after 24 h of infection. In addition, titan cells are resistant to oxygen and nitrogen free radicals of host immune cells and have thick polysaccharide capsule and thickened cell wall that may act as defensive barriers^11^.

It is hypothesized that a large number of titan cells may impede cryptococcal dissemination to the CNS, because their size is a limiting factor for crossing the blood–brain barrier. However, our results show that the large number of titan cells in the lungs of TLR9^−/−^ mice (Fig. 4) does not prevent them from having a greater fungal load in the brain (Fig. 2B). The data presented here provide the first evidence of titan cells in *C. gattii* infection in vivo. Garcia-Barbazan et al., using an experimental infection with *C. neoformans*, showed higher susceptibility of C57BL/6 WT mice in comparison with CD1 WT mice^46^. The C57BL/6 mice had an accentuated survival curve and higher CFUs in the brain, in addition to a higher frequency of titan cells in the lung, as well as a larger diameter these yeasts.

Although caution should be taken in comparing the infection models using *C. neoformans* and *C. gattii*, many aspects appear to be similar. The decrease of IFN-γ and IL-17 levels over time is characteristic of *C. neoformans* infection and was also observed in our model of *C. gattii* experimental infection. The literature has pointed to an increase in the production of IFN-γ in opportunistic mycoses, important in both innate and in adaptive immunity. Some studies show that the increase in the number of IFN-γ-producing CD4^+^ T cells seems to be crucial for the control or delay in the progression of opportunistic mycosis^54,55^. Other studies emphasize the importance of controlling the production of this cytokine, which, at very high levels, ends up being harmful to the host^56^. Therefore, our results, which point to a lower production of IFN-γ in the lungs of infected TLR9^−/−^ mice (Fig. 5A), may highlight this cytokine as a key factor in the control of cryptococcosis, especially at the primary site of infection. C57BL/6 mice infected with the hypervirulent *C. neoformans* H99 strain showed lower levels of IFN-γ after 14 days of infection, as well as higher levels of IL-17, IL-4 and IL-6 in comparison to uninfected C57BL/6 mice^46^. This supports our data, where infected WT mice had low levels of IFN-γ compared to Sham WT mice. However, infected TLR9^−/−^ mice showed even lower levels of cytokine compared to the respective Sham group and the infected WT group.

Similar to IFN-γ, IL-17 appears to play an important role in controlling infection by *Cryptococcus* spp. In a model of infection by *C. neoformans* (B3501 strain) in C57BL/6 IL-17-deficient mice, the role of this cytokine in controlling the disease was identified^35^, mainly due to the activity modulator of GXM. It has also been described that, in a model of infection by *C. neoformans*, a greater infiltration of Th17 lymphocytes was observed in the lungs of C57BL/6 mice, as well as an increased amount of IL-17 in this tissue^57^. Our results show that TLR9-deficient mice infected with *C. gattii* produce less IL-17 in the lungs compared to infected WT mice (Fig. 5B). C57BL/6 mice infected with the *C. neoformans* H99 strain showed an increase in IL-17 levels after 14 days of challenge compared to uninfected mice^46^. Although our data show low levels of IL-17 in the lungs of TLR9^−/−^ mice after 21 days of challenge, it is possible that TLR9 is an important receptor in the protective response against *C. gattii*. Together with the lower levels of IFN-γ in the lungs (Fig. 5A), this data may indicate an immunomodulatory activity of the fungus, where the protective immune response is considerably reduced at the first and principal site of infection.

Previous studies with *C. neoformans* have demonstrated the importance of Th1 and Th17 in host resistance, as well as the role of titan cells in the early stages of infection. We have demonstrated in this work, the susceptibility of C57BL/6 mice to *C. gattii* and the importance of TLR9 to control cryptococcal systemic infection. The absence of TLR9 decreases the host resistance to fungus, probably affecting the early stages of infection. The presence of titan cells and low levels of IFN-γ and IL-17 in the lungs indicate a susceptibility and inability of C57BL/6 mice to control the infection in absence of TLR9.

## Methods

### *Cryptococcus* strain

*Cryptococcus gattii* R265 strain (Serotype B), hypervirulent, VGIIa molecular type, with alpha mating type, was kindly provided by Professor Leonardo Nimrichter (Laboratório de Microbiologia Microbiana, Instituto de Microbiologia Paulo Góes, Universidade Federal do Rio de Janeiro, RJ, Brazil). The cells were cultured in a liquid defined medium (Sabouraud's medium) at 30 °C with continuous shaking (100 rpm) for 4 days and then 5 days in minimal medium^58^.

### Inoculum preparation

Fungal culture (1 mL) was collected and centrifuged at 10,000 rpm for 3 min. The pellet was resuspended in 30 mL sterile PBS and centrifuged twice under the same conditions. The pellet was resuspended once more in 1 mL sterile PBS and counted on a Neubauer chamber. An infection inoculum of 10^4^ yeast in 30 µL PBS was used^42^.

### Mice and infection model

Isogenic mice of the C57BL/6 (WT) and C57BL/6 TLR9^−/−^ (TLR9^−/−^)^59^ strain, male, aged 8–10 weeks, weighing between 25 and 30 g, were used in this study. The C57BL/6 TLR9^−/−^ mice were kindly donated by the Laboratório de Imunofarmacologia, Centro de Ciências da Saúde, Instituto de Biofísica Carlos Chagas Filho, Universidade Federal do Rio de Janeiro, RJ, Brazil, and the C57BL/6 WT lineage was kindly donated by the Instituto de Veterinária, Departamento de Microbiologia e Imunologia Veterinária, Universidade Federal Rural do Rio de Janeiro, RJ, Brazil. The animals were maintained in sterile (grouped) cages, under standardized conditions of temperature (22–23 °C) and light (cycles of 12 h of light and 12 h of dark), commercial feed and drinking water provided ad libitum. The use of the animals in this study was approved by the Ethics Committee on the Use of Animals (CEUA) at UFRJ (Nº: A17/17-061-14). The mice were sacrificed according to the criteria approved by CEUA at the time of the study (2017/2018). All animal work was performed in accordance with Animal Research: Reporting of In Vivo Experiments (ARRIVE) guidelines and regulations.

### Anesthesia and analgesia

Prior to intratracheal infection, anesthesia and analgesia of the animals were performed intraperitoneally with xylazine (10 mg/kg) and ketamine (20 mg/kg) in each animal.

### Intratracheal infection

The animals were subjected to intratracheal infection with 10^4^ encapsulated yeast cells of *C. gattii* (R265 strain) in a total volume of 30 µL/animal, with sterile PBS as the vehicle. Uninfected (Sham) groups were given 30 µL sterile PBS only.

### Animal survival and weight variation

After intratracheal inoculation with 10^4^ yeasts/animal, the mice were monitored every day throughout the course of infection, and any deaths were recorded for the survival analysis up to 80 days or until all the animals died. The animals were weighed before infection (day 0) and on days 7, 14, 21 and 28 after receiving *C. gattii* or PBS only. The masses were plotted on graphs representing the average of the weight variations with the respective standard deviations. For this experiment, 20 WT and 17 TLR9^−/−^ mice were infected; six mice were used as Sham controls for both WT and TLR9^−/−^ groups.

### Determination of fungal load

The organs (lungs, brain and spleen) were collected from euthanized mice at day 21 after infection and completely homogenized in 5 mL sterile PBS in a Petri dish. The total lung homogenate was then diluted 1000× in sterile PBS, while the total brain homogenate was diluted 100×. After this process, 50 μL was spread on a Petri dish containing 2% Sabouraud's agar. The plates were maintained in an incubator at 37 °C and 5% CO_2_. The analysis was performed after 72 h by counting colony forming units (CFU). The absolute values obtained were converted to the logarithmic scale and the statistical differences were analyzed by Student T-test and Mann–Whitney U test. For this experiment, 6 infected WT and 7 infected TLR9^−/−^ mice were used.

### Cytokine dosage

The cytokines of the lung homogenates were measured by the enzyme-linked immunosorbant assays (ELISAs), following the company's protocol (R&D Systems). Briefly, the homogenates were collected as described above (one long in 5 mL of PBS). Harvested supernatants were centrifuged 20 min at 4 °C at 12,000*g* and stored at − 80 °C before, IFN-γ and IL-17 quantification. Elisa plate was coated with anti-IFN-γ and anti-IL-17 overnight, then was blocked with fetal bovine serum and washed five times with PBS-Tween 0.01%. Homogenate were incubated overnight. After was washed 5× with PBS-Tween 0.01%, incubated with anti-IFN-γ or anti-IL-17 with avidin, after wash, it was incubated with streptavidin-HRP, washed again 7× with PBS-Tween 0.01% and 1× with PBS. In the end TMB was added and the reaction was stopped with phosphoric acid. The concentration was determined using a standard curve. The ELISAs were used to determine the production of the cytokines IFN-γ and IL-17. For this experiment, six infected WT and 7 infected TLR9^−/−^ mice were used, as well as 3 Sham WT and 4 Sham TLR9^−/−^ mice.

### Histological sections

After euthanasia, the lungs were excised. Both lungs were placed in identified cassettes and immersed in neutral buffered formalin (Sigma-Aldrich) at 3.7% for 48 h. After fixing the cassettes, they were placed in 70% alcohol until processing. Processing was performed in an automatic processor. Initially, for the diaphanization stage, tissues were transferred to two baths of 100% alcohol. They were then immersed in two xylol baths followed by two baths in liquefied paraffin. Thick cuts of 5 µm of the tissues in paraffin molds were obtained with the aid of a microtome. The sections were submitted to hematoxylin–eosin stains and Alcian Blue, pH 2.5^60^. The slides were deparaffinized and hydrated with distilled water, immersed in hematoxylin for 10 s, washed in running water for 10 min, stained with eosin for 15 s, washed in distilled water, dehydrated, clarified and mounted on Enthelan (Sigma-Aldrich). The slides were analyzed under light microscopy using the Slide Scanner (3D HISTECH). For histological sections, four infected WT and three infected TLR9^−/−^ mice were used; three mice for each Sham group (WT and TLR9^−/−^) were also used.

### Count of titan cells

The counting of yeast cells that were characterized as titan cells was performed using the histological sections of the lungs of infected animals under an optical microscope with a 100× objective lens using immersion oil. Each lung (right and left) was divided into four quadrants. The number of titan cells observed in one hundred yeast cells was determined in each quadrant. The percentage of the respective quadrant was considered an absolute value and an average was obtained. The averages were considered as representative values for each lung. Sections of multiple infected mice (WT and TLR9^−/−^) were analyzed to obtain this result. For titan cell counts, four infected WT and three infected TLR9^−/−^ mice were used; three mice for each Sham group (WT and TLR9^−/−^) were also used.

### Statistical analysis

Statistical analyses were performed using the GraphPad Prism 5.0 program, with the Student T-test with Mann–Whitney U test (as appropriate) for comparison between two groups, log-rank test (Mantel-Cox test) for survival curve analyses or One-Way ANOVA and Two-Way ANOVA with Bonferroni posttest to analyze the weight variation. Values of *P* ≤ 0.05 indicate statistical significance, with significant differences designated as ***P ≤ 0.001, **P ≤ 0.01 and *P ≤ 0.05.
